# Motherhood Role from a Postpartum Perspective: Effects Reflected by High-Risk and Normal Pregnancies

**DOI:** 10.3390/healthcare12131248

**Published:** 2024-06-22

**Authors:** Esra Sarı, Cagri Ates

**Affiliations:** 1 Faculty of Health Science, Department of Midwifery, Van Yuzuncu Yil University, Van 65080, Turkey; 2 Department of Obstetrics and Gynaecology, Van Yuzuncu Yil University, Van 65080, Turkey; cagriates@yyu.edu.tr

**Keywords:** motherhood role, high-risk pregnancies, postpartum, midwife

## Abstract

High-risk pregnancies not only heighten concerns about the baby’s health but also have the potential to impact the mother–infant relationship by shifting the mother’s focus towards her own health needs. This study aims to delve into the intricacies of motherhood roles among women experiencing high-risk pregnancies compared to those with uncomplicated pregnancies, aiming to shed light on the disparities between the two groups. The participants of this descriptive, comparative, and correlational study consisted of literate mothers admitted to a hospital in Eastern Turkey, diagnosed with high-risk pregnancies, and with no prior history of psychological support. Due to an inability to reach the entire population, an unknown sampling method was employed for sampling calculation. The sample comprised 133 mothers with high-risk pregnancies and an equal number of healthy mothers, totaling 266 participants. Data were collected using the “Maternal Introduction Form” and the “Maternal Role Gaining Scale”, and analyses were conducted using the SPSS Statistical Programme. Given the non-normal distribution of the variables, nonparametric tests were applied post reliability analysis. There is a statistically significant difference (*p* < 0.05) in the scores of maternal attitude and anxiety, as well as maternal role and the Maternal Role Gaining Scale, based on various demographic factors such as marriage duration, spouse’s education, family economic status, pre-pregnancy health issues, medication use, hospitalization status and reason, assigned sex of the baby and desired gender, total pregnancies, mode of delivery, postpartum difficulties, support for baby care, feeding method, high-risk pregnancy diagnosis, and week of diagnosis. A Bonferroni corrected analysis also revealed significant differences between mothers with and without high-risk pregnancies.

## 1. Introduction

Motherhood is defined as intrinsic and innate behaviors from a psychoanalytic perspective, whereas sociologists explain the experience of motherhood by investigating the influences of culture, society, and mothers’ real experiences in the process of child-rearing. Therefore, motherhood is shaped by different contexts, perspectives, and roles, becoming a complex concept [1]. Within this complexity, each woman’s transition to motherhood is unique because this experience is influenced by variables associated with the mother, the baby, and the environment [2]. The gaining of the maternal role is a complex and multi-stage process. With each newborn, this role unfolds in four stages: expectations, formal, informal, and personal. Depending on individual differences, the maternal role can be completed within a month or extend up to a year, but typically takes place within an average of four months [3]. A woman’s age, education, physical and mental health, relationship with her partner, significant people in her life, stress, and social support all influence each of these stages, while high-risk pregnancies can further complicate this process [2,3].

High-risk pregnancies are considered a significant public health issue and meeting the healthcare needs in areas where such pregnancies occur is part of the World Health Organization’s Sustainable Development Goal 3 (SDG3) [4]. High-risk pregnancy is associated with unexpected or unforeseen medical or obstetric conditions that pose an actual or potential threat to the health or well-being of the mother or fetus [5]. Globally, more than 20 million women are facing high-risk pregnancies, with 15% of women carrying this risk, resulting in approximately 830 deaths every day. This situation is most prevalent in developing countries, rural areas, and among adolescents [5,6,7]. During pregnancy, high-risk situations can arise either from pre-existing medical conditions or can emerge for the first time alongside pregnancy [6]. Some factors that can make a pregnancy high-risk include high blood pressure, polycystic ovary syndrome, diabetes, kidney diseases, autoimmune diseases, thyroid disorders, obesity, HIV/AIDS, Zika infection, pregnancies under the age of 18 and over the age of 35, alcohol use, tobacco use, or drug use, multiple pregnancies, gestational diabetes mellitus (GDM), preeclampsia, eclampsia, previous preterm birth, fetal congenital anomalies, or genetic conditions [8]. Among these conditions, the most common ones are pregnancy-induced hypertensive disorders (gestational hypertension and preeclampsia), gestational diabetes mellitus (GDM), and acute kidney injury (AKI). Additionally, conditions such as cervical changes, placental abruption, and kidney stones can also occur [6,9]. Moreover, the rising incidence of Placenta Accreta Spectrum due to high rates of cesarean sections is a significant concern for both pregnant women and obstetricians, especially in low-resource settings. The management of this pathology, whether through conservative management of the uterus or cesarean hysterectomy, remains controversial worldwide [10]. When pregnancy is associated with these complications, it becomes a stressful period filled with potential physical, psychological, and socio-economic consequences for women at high risk [5]. It was stated that mothers in high-risk pregnancies experience anxiety and fear about the survival and healthy development of their babies [2,11]. Expectant mothers often experience the excitement of becoming a mother while also grappling with the fear of harm to themselves and their baby. High-risk pregnancies intensify these feelings, potentially obstructing goals of having a healthy baby or achieving a healthy delivery due to factors such as illness, separation, and imposed restrictive rules. Consequently, expectant mothers simultaneously navigate the excitement of impending motherhood and contend with the risks and challenges of pregnancy-related illnesses [2].

This study aims to identify the maternal role of women experiencing high-risk pregnancies and those who are not, as well as to elucidate the differences between the two groups. The following research questions were addressed: (1) How do women experiencing high-risk pregnancies perceive their maternal role during the postpartum period? (2) How do women experiencing healthy pregnancies perceive their maternal role during the postpartum period?

## 2. Materials and Methods

This study was conducted in a descriptive, causal-comparative, and correlational manner.

### 2.1. Population

The population of this study consisted of mothers aged 18–49 who visited a university hospital in the eastern part of Turkey for any reason between February and May 2024, was diagnosed with high-risk pregnancies or having a healthy pregnancy, had never received any psychological support before, and were literate. Since it was not possible to reach the entire population in the sample calculation, this study employed the method of sampling from an unknown population [12].

$$n=\frac{t²\times p\times q}{t²}$$

n = Number of individuals to be sampled;
p = The frequency of occurrence of the phenomenon under study;
q = The frequency of non-occurrence of the phenomenon under study (1 − p);
*t* = The theoretical value found in the t-table at a specific degree of freedom and determined level of significance;
p = The probability of occurrence of the phenomenon under study, 0.06;
q = The probability of non-occurrence of the phenomenon under study, 0.94;
N = The estimated number of individuals in the population, 5000;
d = The desired deviation based on the frequency of occurrence of the phenomenon, 0.04.

**Figure 1:**
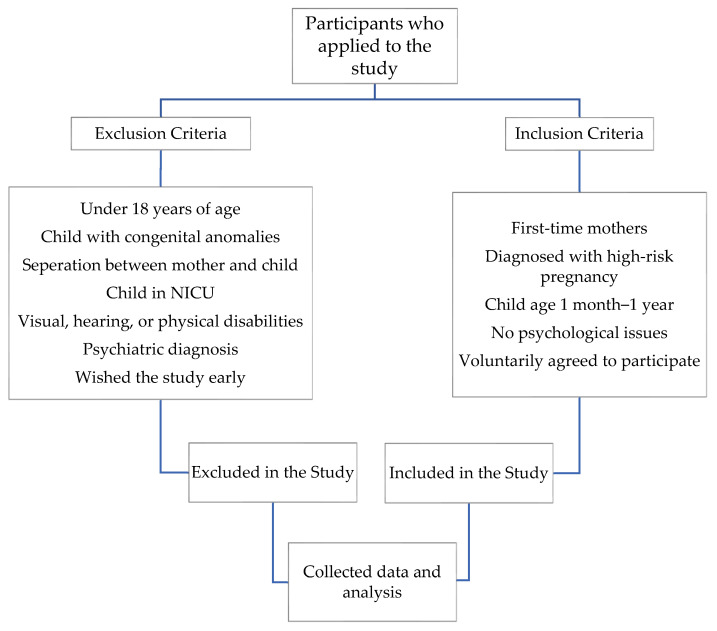
The study flowchart.

According to the theoretical value of 1.96 obtained from the formula at a specific degree of freedom and determined level of significance, the research sample reached 266 mothers, including 133 diagnosed with high-risk pregnancies and 133 healthy mothers (control group), calculated using n = (1.96)^2^ × 0.06× 0.94/(0.04)^2^. The inclusion and exclusion criteria of the study are clearly outlined in Figure 1.

### 2.2. Data Collection

Data collection was entirely conducted face-to-face. Data were collected using the “Maternal Introduction Form” and the “Maternal Role Gaining Scale”.

Maternal Introduction Form: Consistent with the study’s aim, the questionnaire comprised 28 questions following the literature. The survey included socio-demographic characteristics such as age, education level, and duration of marriage, as well as high-risk pregnancy diagnoses; other complaints encountered during pregnancy; and obstetric characteristics such as parity, gravidity, and abortions.

Maternal Role Gaining Scale: The scale developed by Arpacı Kızıldağ and Yiğit in 2022 comprised 26 items and 3 subscales, has been validated. The subscale “Maternal Attitude” included 11 items, “Maternal Anxiety” 7 items, and “Maternal Role” 8 items. The Cronbach’s Alpha coefficient of the scale was found to be 0.793. The scale was designed using a 5-point Likert scale (1. Strongly Disagree, 2. Disagree, 3. Neither Agree nor Disagree, 4. Agree, 5. Strongly Agree). Each item on the scale was scored as 1-2-3-4-5. Items 7, 8, 9, 10, 14, 15, 19, 20, 21, 22, 23, 24, 25 on the scale were reverse-coded, meaning they were scored as 5-4-3-2-1. The maximum score obtainable from the scale was 130, and the minimum score was 26, with increasing scores indicating the gaining of the maternal role [13].

### 2.3. Data Analysis

The data obtained in the research were analyzed using the free trial version of IBM SPSS Statistics (IBM SPSS for Windows version 25, IBM Corporation, Armonk, NY, USA, 2017). A “reliability analysis” (Cronbach’s Alpha) was conducted to test the reliability of the scales used. Descriptive statistical methods (number, percentage, minimum–maximum values, median, mean, and standard deviation) were employed in evaluating the data.

It was investigated whether the measurement instruments exhibited a normal distribution according to the Kolmogorov–Smirnov test. Based on this, it was concluded that the variables did not follow a normal distribution, and nonparametric tests were used for evaluation. The Mann–Whitney U test was employed for comparing differences between two independent groups, while the Kruskal–Wallis test was utilized for comparing more than two independent groups.

## 3. Results

### Demographic Results

The mean age of the 266 participating mothers was 31.26 ± 3.95 years; 48.1% of the mothers had been married for 4–6 years, with the majority (80.4%) being employed; and 90.6% had a university degree or higher. Mothers’ socio-demographic characteristics are presented in Table 1.

According to the obstetric characteristics of the mothers: 86.1% had experienced one pregnancy, while 13.9% had experienced two pregnancies; 86.9% of the pregnancies were planned, and all of them attended doctor visits for pregnancy monitoring; 94% answered “no” to the question regarding pre-pregnancy health issues, while 90.6% answered “no” to the question regarding medication usage during pregnancy; and examining the distribution based on “hospitalization during pregnancy”, 32.6% answered “yes”, while 67.4% answered “no”. The obstetric characteristics of the mothers are provided in Table 2.

The reliability analysis results of the scale used in the research are provided in Table 3.

The results of the normality analysis of the dimensions of the scale used in the research are provided in Table 4.

The mean of the Maternal Attitude dimension was 46.72, the mean of the Maternal Anxiety dimension was 24.66, the mean of the Maternal Role dimension was 28.48, and the mean of the Maternal Role Gaining Scale was 99.87. Descriptive statistics for the Maternal Role Gaining Scale are provided in Table 5.

According to demographic characteristics such as mothers’ marital duration, spouses’ educational level, family economic status, pre-pregnancy health issues, medication usage, hospitalization, reason for hospitalization, baby’s gender, and desired gender, there was a statistically significant difference between the scores of the maternal attitude dimension and maternal anxiety dimension (*p* < 0.05). Additionally, there was a statistically significant difference between the maternal role dimension and the Maternal Role Gaining Scale according to factors such as mothers’ marital duration, education level, spouses’ education level, family economic status, total number of pregnancies, pre-pregnancy medication usage, hospitalization, reason for hospitalization, method of delivery, baby’s desired gender, experiencing difficulties related to the baby in the postpartum period, receiving support with baby care, feeding method for the baby, diagnosis of high-risk pregnancy, and the week of detecting risk factor (*p* < 0.05). A comparison of scale and dimension scores according to demographic characteristics of participating mothers is provided in Table 6.

## 4. Discussion

The postpartum period can be a joyful experience in women’s lives, but it can also be filled with challenges as new mothers adapt to their maternal role. In this study conducted with the participation of 266 mothers from Turkey, the impact of mothers’ socio-demographic and obstetric characteristics on maternal attitude, anxiety, and role was examined, and the results addressed the research questions (Q1 and Q2). 

The study findings revealed that the mean age of the mothers was 31.26 ± 3.95 years, with the majority (80.4%) being employed. The vast majority of participants (90.6%) had a university degree or higher. Mucuk et al. (2016) demonstrated in their study that maternal education level affected the maternal role [14]. Similarly, in the study by Koç et al. (2016), it was found that the education level of mothers positively influenced the development of the maternal role [14]. In another study, it was observed that as parents’ education levels increased, their preparation levels for the roles of mother and father also increased [3]. Mothers with higher education levels may acquire more knowledge about baby care topics and conduct research, thereby increasing their self-confidence during the prenatal period and feeling more competent and secure in baby care. Additionally, these mothers may adapt better to the maternal role and effectively cope with the challenges encountered during the process.

In the study, it was found that socio-demographic factors such as mothers’ marital duration, education levels, spouses’ education levels, and family economic status had a significant impact on maternal attitude and anxiety (*p* < 0.05). However, it was observed that mothers’ employment status did not significantly affect the maternal role (*p* > 0.05). Nevertheless, in another study, it was found that mothers’ employment status positively influenced maternal role gaining [15], and women with higher income levels adapted better to pregnancy and motherhood compared to those with moderate- or low-income levels [16]. This suggests that mothers with higher education and income levels may be better equipped with more resources and support, have easier access to information, and therefore adapt better to the maternal role. In contrast to our findings, it is believed that the skills gained by working mothers in their professional lives and their structured routines help them adapt more effectively to the maternal role.

In evaluations based on obstetric characteristics, it was determined that factors such as total number of pregnancies, pre-pregnancy medication usage, and hospitalization status had significant effects on maternal role and the Maternal Role Gaining Scale (*p* < 0.05). Additionally, it was found that participants who had a vaginal birth had higher mean scores on the maternal role scale compared to those who had a cesarean section. However, there was no statistically significant difference in terms of planned or unplanned pregnancy for the Role Gaining Scale (*p* > 0.05). Contrary to our study, Uçar and Özkan (2023) suggested that women who planned their pregnancies might have influenced their perceptions of babies’ roles [17]. These results indicate that mothers’ experiences during pregnancy and childbirth play a significant role in how they perceive and adopt their maternal roles. This, however, implies that the findings of different studies can be in conflict with one another and point to the need for further research in this field.

This study also demonstrated that the feeding method of the baby and receiving support with baby care were associated with maternal role. Postnatal depression is an important issue in postpartum course [18]. Islam et al. (2021) stated in their study on postpartum depression (PPD) that the likelihood of experiencing PPD was 7.58 times higher for mothers who did not “only breastfeed” their babies compared to those who did “only breastfeeding”. Additionally, it was found that maternal stress and social support significantly increased the likelihood of PPD in mothers who discontinued only breastfeeding early and had high stress with limited social support [19]. Furthermore, it was found that mothers who received postpartum baby care and feeding support embraced their maternal roles more positively. Particularly, social support systems were shown to be critically important in adapting to the maternal role. In Kim’s (2021) study with primiparous mothers, it was found that social support had a significant regulatory effect only on the perception of maternal role [20]. This suggests that receiving social support and assistance with baby care may contribute to mothers embracing their maternal roles more positively. 

In this study, it was found that specific obstetric conditions such as the diagnosis of high-risk pregnancy and the week when this diagnosis was received led to significant differences in maternal role (*p* < 0.05). In the literature, it was noted that preterm labor disrupts preparation for the maternal role [2], and adverse symptoms during pregnancy such as bleeding could interrupt preparations and make bonding with the fetus difficult [21]. It was observed that pregnant women hospitalized due to early membrane rupture experienced intense stress, mothers who gave birth prematurely did not feel like mothers until they saw and touched their babies, and their maternal role competence was low [2]. Additionally, previous pregnancy losses were noted to make it difficult to accept the current pregnancy and bond with the fetus [22,23]. These findings suggest that high-risk pregnancies may pose additional challenges in the process of embracing the maternal role and that mothers may require more support and guidance.

This study demonstrated that mothers’ socio-demographic and obstetric characteristics had significant effects on maternal attitude, anxiety, and role. It was found that the diagnosis of high-risk pregnancy created a statistically significant difference in the sub-dimensions of maternal role and the total mean scores of the Maternal Role Gaining Scale (*p* < 0.05). According to the corrected Bonferroni test, it was observed that the mean scores of the sub-dimensions of maternal role for participants without a diagnosis of high-risk pregnancy were higher than those with diagnoses of hypertension and placenta previa. The mean scores of the sub-dimensions of maternal role for participants diagnosed with gestational diabetes were also higher compared to those with diagnoses of hypertension, placenta previa, and preeclampsia. Additionally, it was found that the total mean scores of the maternal role scale for participants without a diagnosis of high-risk pregnancy were higher compared to those diagnosed with gestational diabetes, hypertension, and placenta previa. Statistically significant differences were also found based on the weeks when risk factors were detected, with participants without a diagnosis of high-risk pregnancy having higher mean scores compared to those diagnosed after 28 weeks. It was observed that mothers experiencing high levels of stress and anxiety, physical and health problems, lack of social support, and inadequate psychological preparation had lower mean scores in the sub-dimensions of maternal role and total mean scores of the maternal role scale.

## 5. Conclusions

Mothers experiencing a high-risk pregnancy may face additional psychosocial challenges, making their adjustment to the maternal role more complex, and the transition to parenthood can impact mothers both physically and mentally. In such cases, a careful approach is required. Providing a supportive environment for mothers to adapt to new responsibilities and relationships is crucial. This entails offering both emotional and practical support, thus highlighting the significant role of midwives in the postpartum period. Providing prenatal and postnatal education to reduce mothers’ anxiety levels and prepare them for motherhood and welcoming the newborn is essential. Additionally, providing support and comfort to mothers during childbirth and ensuring a comfortable birthing environment are necessary. These approaches can help mothers cope with psychological distress such as depression, anxiety, and insomnia and support their adaptation to the maternal role.

## 6. Strengths and Limitations 

This study comprehensively elucidated the impact of mothers’ socio-demographic and obstetric characteristics on their maternal attitude, anxiety, and role. However, the limitation of the study to participants from Turkey restricts the generalizability of the findings to other cultures and societies. Future research is recommended to enhance the generalizability and validity of the findings by conducting similar studies in different cultural and social contexts. Additionally, longitudinal studies are needed to delve deeper into the changes in maternal attitude, anxiety, and role over time.

Furthermore, there are no data on estimated blood loss, postpartum hemorrhage, anemia, blood transfusion, infection, and antibiotic therapy use. These factors could potentially influence the outcomes and should be considered in future research.

Due to the absence of the Maternal Role Gaining Scale in the literature, this section included discussions on alternative maternal role scales. Moreover, the Maternal Role Gaining Scale used in the study has not been tested or validated for reliability and validity in different settings or by independent sources outside of this particular study.

## Figures and Tables

**Table 1:** Mothers’ socio-demographic characteristics (n = 266).

Characteristics		n	%
Age ($\bar{X}$ ± SS, 31.26 ± 3.95)	≤31 years	123	46.2
	≥31 years	143	53.8
The duration of marriage (years) ($\bar{X}$ ± SS, 5.02 ± 2.54)	1–3 years	71	26.7
	4–6 years	128	48.1
	≥7 years	67	25.2
The age of the spouse ($\bar{X}$ ± SS, 33.76 ± 3.88)	≤34 years	134	50.3
	≥34 years	132	49.7
Employment Status	Yes	214	80.4
	No	52	19.6
Spouse’s employment Status	Yes	266	100.0
Educational status	Secondary school graduate	25	9.4
	High school graduate or above	241	90.6
Spouse’s educational status	Secondary school graduate	65	24.4
	High school graduate or above	201	75.6
Family type	Nuclear family	266	100.0
Family economic status	Below medium	10	3.7
	Medium	178	67.0
	Above medium	78	29.3

**Table 2:** The obstetric characteristics of the mothers (n = 266).

Total number of pregnancies	One	230	86.4
	Two	36	13.6
Planned pregnancy status	Planned	231	86.8
	Unplanned	35	13.2
Attendance to doctor visits for pregnancy monitoring during pregnancy	Yes	266	100.0
Presence of any pre-existing health issues before pregnancy	Yes	16	5.1
	No	250	93.9
Presence of any medication used before and currently during pregnancy	Yes	25	9.4
	No	241	90.6
Hospitalization during this pregnancy	Yes	87	32.8
	No	179	67.2
Reason for hospitalization	Pre-eclampsia	51	19.1
	Hypertension	13	4.9
	Gestational diabetes	23	8.7
	Not hospitalized	179	67.3
Method of delivery	Normal delivery	56	21.0
	Cesarean section	210	79.0
Assigned sex of the baby	Girl	166	62.4
	Boy	100	37.6
Desired gender of the baby	Yes	254	95.5
	No	12	4.5
Receiving any information about baby care during the prenatal period	Yes	242	91.0
	No	24	9.0
Receiving support from family members during labor and the postpartum period	Yes	252	94.7
	No	14	5.3
Experiencing any difficulties in the postpartum period	Yes (fatigue, pain, etc.)	10	3.8
	No	256	96.2
Experiencing any difficulties related to the baby in the postpartum period	Yes	85	32.0
	No	181	68.0
Receiving support with baby care	Yes	192	72.2
	No	74	27.8
Seeking medical care for both the baby and oneself for postpartum monitoring	Yes	266	100.0
Feeding method for the baby	Breastfeeding only	215	80.8
	Formula feeding only	7	2.6
	Solid food only	7	2.6
	Breast milk + Formula	10	3.8
	Breast milk + Solid food	9	3.4
	Formula + Solid food	9	3.4
	Breast milk + Formula + Solid food	9	3.4
Diagnosis of high-risk pregnancy	Not at risk	133	50.0
	Gestational diabetes	46	17.3
	Hypertension	26	9.8
	Placenta previa	16	6.0
	Preeclampsia	45	16.9
Week of detecting risk factor	Not at risk	133	50.0
	≤28 week	33	12.4
	>28 week	100	37.6

**Table 3:** Reliability analysis results of the Maternal Role Gaining Scale (n = 266).

Scale and Dimensions	Cronbach’s Alpha
Maternal Attitude Dimension	0.886
Maternal Anxiety Dimension	0.795
Maternal Role Dimension	0.700
Maternal Role Gaining Scale	0.757

**Table 4:** Normality analysis results of the Maternal Role Gaining Scale (n = 266).

Scale and Dimensions	Kolmogorov–Smirnov		Status
	Statistics	*p*	
Maternal Attitude Dimension	0.218	0.000	Not normal
Maternal Anxiety Dimension	0.196	0.000	Not normal
Maternal Role Dimension	0.234	0.000	Not normal
Maternal Role Gaining Scale	0.234	0.000	Not normal

**Table 5:** Descriptive statistics for the Maternal Role Gaining Scale (n = 266).

Scale and Dimensions	Minimum	Maximum	Mean	SD
Maternal Attitude Dimension	14.00	54.00	46.72	7.39
Maternal Anxiety Dimension	11.00	34.00	24.66	4.13
Maternal Role Dimension	21.00	33.00	28.48	3.23
Maternal Role Gaining Scale	62.00	113.00	99.87	9.41

**Table 6:** Comparison of scale and dimension scores according to demographic characteristics of participating mothers.

Variables		Maternal Attitude Dimension						Maternal Anxiety Dimension					
		Med	$\bar{X}$	SD	Test Value	*p*	Bonferroni	Med	$\bar{X}$	SD	Test Value	*p*	Bonferroni
Age	≤31	48.00	45.16	9.94	−0.688 ^m^	0.491		25.00	24.57	4.67	−0.527 ^m^	0.598	
	≥31	48.00	48.06	3.64				25.00	24.74	3.62			
Marital duration (year)	1–3 year (1)	48.00	43.1	11.89	12.159 ^k^	0.002 *	2 > 1	25.00	23.9	5.44	9.593 ^k^	0.008 *	2 > 1
	4–6 year (2)	50.00	48.45	4.45			2 > 3	26.00	25.27	3.38			
	≥7 year (3)	47.00	47.24	3.36				25.00	24.28	3.72			
Spouse’s educational level	Secondary school graduate	48.00	44.86	11.24	−0.157 ^m^	0.875		22.00	23.29	4.82	−4.753 ^m^	0.000 *	
	High school graduate or above	48.00	47.32	5.52				26.00	25.1	3.79			
Family economic status	Below medium (1)	46.00	48.4	3.13	1.782 ^k^	0.41		16.00	18.4	3.47	20.271 ^k^	0.000 *	2 > 1
	Medium (2)	48.00	45.99	8.47				25.00	24.65	4.31			3 > 1
	Above medium (3)	50.00	48.19	4.21				26.00	25.49	2.97			
Presence of any pre-existing health issues before pregnancy	Yes	49.50	35.13	19.28	−0.754 ^m^	0.451		28.00	29.94	3.8	−4.440 ^m^	0.000 *	
	No	48.00	47.46	5.16				25.00	24.32	3.92			
Presence of any medication used before and currently during pregnancy	Yes	42.00	37.6	15.61	−2.959 ^m^	0.003 *		28.00	29.24	3.15	−5.510 ^m^	0.000 *	
	No	48.00	47.67	5.14				25.00	24.19	3.93			
Hospitalization status during this pregnancy	Yes	46.00	46.77	3.96	−2.677 ^m^	0.007 *		25.00	23.85	3.26	−3.190 ^m^	0.001 *	
	No	49.00	46.7	8.58				26.00	25.05	4.45			
Reason for hospitalization	Preeclampsia (1)	46.00	46.16	3.4	13.260 ^k^	0.004 *	4 > 1	25.00	23.92	3.08	10.462 ^k^	0.015 *	4 > 1
	Hypertension (2)	52.00	48.15	5.27				21.00	23.38	3.48			
	Gestational diabetes (3)	48.00	47.35	4.2				25.00	23.96	3.65			
	Not hospitalized (4)	49.00	46.7	8.58				26.00	25.05	4.45			
	Cesarean section (5)	48.00	46.26	8.02				25.00	24.41	4.42			
Assigned sex of the baby	Girl	49.00	46.73	8.33	−2.146 ^m^	0.032 *		26.00	24.73	4.64	−1.346 ^m^	0.178	
	Boy	47.00	46.71	5.53				25.00	24.54	3.15			
Desired gender of the baby	Yes	48.00	46.55	7.49	−2.298 ^m^	0.022 *		25.00	24.67	4.21	−0.591 ^m^	0.554	
	No	50.00	50.33	3.03				25.00	24.5	1.57			
Receiving support from family members during labor and the postpartum period	Yes	48.00	46.82	7.52	−2.446 ^m^	0.014 *		25.00	24.62	4.14	−0.960 ^m^	0.337	
	No	47.00	45.07	4.3				26.00	25.33	4.03			
Experiencing any difficulties related to the baby in the postpartum period	Yes	46.00	44.42	6.21	−6.474 ^m^	0.000 *		25.00	22.95	4.64	−4.212 ^m^	0.000 *	
	No	51.00	47.8	7.66				26.00	25.46	3.61			
Feeding method for the baby	Breastfeeding only (1)	48.00	46.73	7.46	13.950 ^k^	0.030 *	7 > 2	25.00	24.93	3.98	26.820 ^k^	0.000 *	1 > 2
	Formula feeding only (2)	25.00	34.71	12.39				11.00	16	6.35			3 > 2
	Solid food only (3)	41.00	45.29	5.56				29.00	26.86	3.08			1 > 7
	Breast milk + Formula (4)	51.00	49.3	3.74				25.50	25.1	2.64			3 > 7
	Breast milk + Solid food (5)	47.00	48.22	2.44				26.00	25.44	1.67			
	Formula + Solid food (6)	48.00	48.4	1.26				26.00	25.4	1.58			
	Breast milk + Formula + Solid food (7)	51.00	50.89	1.27				20.00	21.22	2.44			
Diagnosis of high-risk pregnancy	Not at risk (0)	51.00	48.91	3.4	22.048 ^k^	0.000 *	0 > 3	26.00	25.32	3.51	11.943 ^k^	0.018 *	0 > 3
	Gestational diabetes (1)	48.00	47.11	6.4			0 > 4	25.50	24.17	3.71			
	Hypertension (2)	43.00	42.27	13.08				22.00	25.08	4.69			
	Placenta previa (3)	34.00	35.94	14.78				23.50	20.69	8.44			
	Preeclampsia (4)	46.00	46.22	3.51				25.00	24.36	2.74			
Week of detecting risk factor	Not at risk (0)	51.00	48.91	3.4	16.749 ^k^	0.000 *	0 > 2	26.00	25.32	3.51	17.152 ^k^	0.000 *	0 > 1
	≤28 week (1)	48.00	43.52	10.99				21.00	21.45	5.87			2 > 1
	>28 week (2)	46.00	44.85	8.86				25.00	24.83	3.76			
**Variables**		**Maternal Role Dimension**						**Maternal Role Gaining Scale**					
		**Med**	$\bar{X}$	**SD**	**Test Value**	***p***	**Bonferroni**	**Med**	$\bar{X}$	**SD**	**Test Value**	***p***	**Bonferroni**
Age	≤31	29.00	28.63	2.86	−0.450 ^m^	0.653		101.00	98.37	11.11	−1.692 ^m^	0.091	
	≥31	29.00	28.35	3.53				102.00	101.15	7.48			
Marital duration (year)	1–3 year (1)	28.00	27.93	3.21	24.842 ^k^	0.000 *	3 > 1	99.00	94.93	12.62	19.524 ^k^	0.000 *	2 > 1
							3 > 2						3 > 1
	4–6 year (2)	29.00	28.05	3.39				102.00	101.77	8.14			
	≥7 year (3)	30.00	29.91	2.49				101.00	101.43	4.89			
Educational level	Secondary school graduate	30.00	29.88	1.94	−2.427 ^m^	0.015 *		102.00	102.16	3.88	−0.369 ^m^	0.712	
	High school graduate or above	29.00	28.34	3.31				102.00	99.63	9.78			
Spouse’s educational level	Secondary school graduate	28.00	27.46	3.24	−4.043 ^m^	0.000 *		98.00	95.62	8.74	−5.993 ^m^	0.000 *	
	High school graduate or above	29.00	28.81	3.17				102.00	101.23	9.23			
Family economic status	Below medium (1)	33.00	31.3	2.21	16.664 ^k^	0.000 *	1 > 2	95.00	98.1	4.56	4.710 ^k^	0.095	
	Medium (2)	29.00	28.81	3.01			1 > 3	102.00	99.45	10.19			
	Above medium (3)	29.00	27.37	3.47			2 > 3	102.00	101.05	7.84			
Total number of pregnancies	One	29.00	28.13	3.24	−4.605 ^m^	0.000 *		101.00	99.3	9.8	−2.343 ^m^	0.019 *	
	Two	32.00	30.68	2.15				102.00	103.38	5.38			
Presence of any medication used before and currently during pregnancy	Yes	32.00	30.48	2.49	−2.821 ^m^	0.005 *		103.00	97.32	13.96	−0.844 ^m^	0.398	
	No	29.00	28.28	3.24				102.00	100.13	8.81			
Hospitalization status during this pregnancy	Yes	29.00	28.06	3.93	−0.060 ^m^	0.952		101.00	98.68	6.55	−3.300 ^m^	0.001 *	
	No	29.00	28.69	2.83				102.00	100.44	10.49			
Reason for hospitalization	Preeclampsia (1)	29.00	27.02	4.17	11.103 ^k^	0.011 *	3 > 1	99.00	97.1	6.95	18.109 ^m^	0.000 *	4 > 1
	Hypertension (2)	29.00	28.46	3.55				102.00	100	6.43			
	Gestational diabetes (3)	31.00	30.13	2.62				102.00	101.43	4.56			
	Not hospitalized (4)	29.00	28.69	2.83				102.00	100.44	10.49			
Method of delivery	Normal delivery	29.00	28.68	2.89	−0.145 ^m^	0.884		103.00	102.73	5.04	−1.992 ^m^	0.046 *	
	Cesarean section	29.00	28.43	3.32				101.00	99.1	10.14			
Desired gender of the baby	Yes	29.00	28.57	3.24	−2.330 ^m^	0.020 *		102.00	99.79	9.52	−0.277 ^m^	0.782	
	No	26.00	26.67	2.46				99.00	101.5	6.75			
Experiencing any difficulties related to the baby in the postpartum period	Yes	30.00	28.15	4.01	−0.527 ^m^	0.598		99.00	95.53	11.12	−5.648 ^m^	0.000 *	
	No	29.00	28.64	2.8				102.00	101.89	7.74			
Receiving support with baby care	Yes	29.00	28.19	3.14	−3.278 ^m^	0.001 *		101.00	99.35	10.2	−0.393 ^m^	0.694	
	No	29.00	29.24	3.37				102.00	101.19	6.9			
Feeding method for the baby	Breastfeeding only (1)	29.00	28.36	3.23	28.525 ^k^	0.000 *	5 > 2	101.00	100.01	8.65	20.281 ^k^	0.002 *	1 > 2
	Formula feeding only (2)	26.00	25.57	2.37			6 > 2	62.00	76.29	18.45			4 > 2
	Solid food only (3)	32.00	30.14	2.67			5 > 7	102.00	102.29	2.93			5 > 2
	Breast milk + Formula (4)	29.00	27.5	4.28			6 > 7	105.00	101.9	9.77			6 > 2
	Breast milk + Solid food (5)	32.00	31.33	1.32				105.00	105	1.5			
	Formula + Solid food (6)	32.00	31	2.31				106.00	104.8	2.7			
	Breast milk + Formula + Solid food (7)	28.00	27.78	1.09				99.00	99.89	3.06			
Diagnosis of high-risk pregnancy	Not at risk (0)	29.00	29.23	2.42	24.475 ^k^	0.000 *	0 > 2	103.00	103.46	4.9	40.464 ^k^	0.000 *	0 > 1
	Gestational diabetes (1)	29.50	29.3	3.29			0 > 3	102.00	100.59	7.01			0 > 2
	Hypertension (2)	28.50	26.73	3.88			1 > 2	100.00	94.08	10.96			0 > 3
	Placenta previa (3)	26.00	26.94	2.49			1 > 3	81.00	83.56	19.86			
	Preeclampsia (4)	29.00	26.98	4.03			1 > 4	99.00	97.56	6.96			
Week of detecting risk factor	Not at risk (0)	29.00	29.23	2.42	12.070 ^k^	0.002 *	0 > 2	103.00	103.46	4.9	30.619 ^k^	0.000 *	0 > 1
													0 > 2
	≤28 week (1)	29.00	29.09	2.8				100.00	94.06	16.54			
	>28 week (2)	29.00	27.28	3.91				101.00	96.96	8.95			

Notes: * *p* < 0.05, ^m^ Mann–Whitney U test, ^k^ Kruskal–Wallis test.

## Data Availability

The datasets used and analyzed during the current study are available from the corresponding author on reasonable request.
